# Trends in the market for drug delivery devices categorized as combination drugs and medical devices and regulatory challenges for autoinjectors in Japan

**DOI:** 10.3389/fmedt.2024.1461460

**Published:** 2024-08-06

**Authors:** Makiko Mochizuki, Hideki Maeda

**Affiliations:** Department of Regulatory Science, Graduate School of Pharmaceutical Science, Meiji Pharmaceutical University, Tokyo, Japan

**Keywords:** combination products, combination drugs, autoinjectors, prefilled syringes, Japan, medical devices, drug device combination, drug delivery devices

## Abstract

**Background:**

Although a variety of drug delivery devices have been launched in recent years, few studies have comprehensively investigated the market trends of combination drugs and medical devices approved or certified in Japan and the regulatory challenges related to their approval. Among the drug delivery devices, autoinjectors are more convenient than traditional prefilled syringes and are designed with safety features to prevent needlestick accidents, allowing self-injection by patients. Therefore, autoinjectors have been incorporated into the treatment of various diseases and have shown significant growth among drug delivery devices.

**Aim:**

This study aimed to investigate the market trends of combination drugs approved in Japan, especially those with autoinjector formulations, and to explore the challenges in the regulatory aspects of combination drugs.

**Methods:**

Information on the number of marketed drugs and medical devices was obtained from the Pharmaceuticals and Medical Devices Agency (PMDA) database using specific definitions. We looked at the annual changes in the number of drug delivery devices approved and certified as combination drugs or medical devices and the number of canceled certifications. We also examined the classification and main certification criteria for Japanese medical device nomenclature.

**Results:**

The study suggested that the number of combination drugs with autoinjector formulations is increasing, replacing previously approved or certified pen-type medication injectors. Moreover, 53% of all drug products were approved for autoinjector formulations after the initial authorization approval in Japan, and more than half of them obtained approval for additional formulations for autoinjectors within five years of the initial authorization approval, with the largest number of cases obtaining approval for additional formulations two years later.

**Conclusion:**

The lack of clear regulatory requirements for autoinjectors may lead to confusion among applicants. Furthermore, there are challenges in filing regulatory applications, thus hindering the rapid launch of combination drug-utilizing devices with superior usability.

## 1 Introduction

In recent years, diversification, complexity, and advancements in combination products that combine drugs, medical devices, and cellular and tissue-based products have progressed in Japan. Among these, the combination drug market, which consists of combinations of drugs, and medical devices and where the primary mode of action is attributed to the drugs, has shown significant growth. This growth has been driven by a variety of factors, including the growth of biologics, improvement in product-added value, and the shift from inpatient to outpatient treatment, which have led to research and development efforts aimed at improving safety, efficacy, and user convenience (1). The development of various drug delivery devices, such as pen injectors, autoinjectors, and on-body injectors, which are more user-friendly and offer higher functionality than conventional pre-filled syringes, is considered valuable in reducing patient burden (2). Autoinjectors, which are more convenient and designed with safety features to prevent needlestick accidents compared to traditional prefilled syringes, allow self-injection by patients and have been incorporated into the treatment of various diseases (3–6).

In Japan, drug delivery devices include cases in which the device component is approved or certified as a medical device, the cartridge attached to the device is approved as a pharmaceutical product, and the pharmaceutical product is integrated with the device as a prefilled drug.

The U.S. FDA defines combination products as “A product comprised of two or more regulated components, i.e., drug/device, biologic/device, drug/biologic, or drug/device/biologic, that are physically, chemically, or otherwise combined or mixed and produced as a single entity” (7). On the other hand, the definition of combination products in Japan is as follows: “products marketed as a single drug, medical device, or cellular and tissue-based product that combine two or more types of drug, device, processed cell, etc. that are expected to fall under the category of drugs, medical devices, or cellular and tissue-based products if marketed individually” (8). A key difference from the U.S. definition is that biological products are included in the definition of drugs in Japan, and cellular and tissue-based products serve as a third element instead of biological products. Furthermore, different regulatory review bodies are involved depending on which of the three elements a combination product falls under.

Combination products, which are combinations of drugs and medical devices, are in between the regulations for drugs and medical devices; only a few regulatory notifications on combination products have been issued by health authorities, and clear pharmaceutical requirements have not been established. Moreover, because of the nature of combination drugs, which require approval as drugs, there are no standardized rules for packaging formats or naming conventions, nor is there much information related to the administration devices included in the filing of review reports (9). Owing to these factors, it is difficult to grasp the overall picture of combination drugs and the medical device market for drug delivery devices in Japan.

Few studies have comprehensively investigated Japan's combination drug and medical device markets for drug delivery devices and the challenges involved in regulatory applications. This study aimed to investigate the number of drug delivery devices approved or certified as combination drugs and medical devices in Japan, focusing on autoinjectors, which have shown significant growth. In addition, this study aims to examine the challenges in regulatory applications.

## 2 Method and materials

### 2.1 Number of drug delivery devices as combination drugs in the Japanese market

Based on the Pharmaceuticals and Medical Devices Agency's (PMDA) information search database (10) (as of June 3, 2024), the number of marketed products was determined using the number of package inserts for each combination drug. The search was conducted by looking for package inserts that included device names, such as “syringe,” “pen,” and “autoinjector,” in the “packaging” section. The functionality of each product was investigated using review reports, interview forms, and user guides. This study focused on products that have obtained approval as drugs, excluding those not intended for therapeutic purposes, such as diagnostic products. And biosimilars and generic drugs are included. If a brand has several different doses, they are counted as one product.

Definitions for search:

• Prefilled syringes: Prefilled devices are injected directly into the body, such as by subcutaneous or intramuscular injection, through the attachment of a needle.
• Pen injectors: Needle-free disposable devices for multiple administrations
• Autoinjectors: Single-use disposable devices with needles for single administration
• On-body injectors (11): Active devices applied to the abdomen or other body parts.

### 2.2 Investigation of autoinjectors in the Japanese market

The investigation further explored the autoinjectors identified in Section 2.1 using interview forms and other resources. This study examined the approval years of autoinjectors since 2000, therapeutic classification names, the proportion of approvals for device formulations other than autoinjectors, the proportion of approvals obtained through the addition of new formulations, and the number of years between the initial marketing approval and approval of the autoinjector formulation. In cases where a product had multiple therapeutic classifications, each classification was counted separately.

### 2.3 Annual changes in the number of drug delivery devices approved and certified as medical devices and the number of canceled certifications

Using the PMDA's Medical Device Information Search Site (12) (as of June 9, 2024) and the certification product list (up to the items certified in March 2024), this study investigated the number of approved and certified medical devices for reusable medication/vaccine injectors, pen-type medication injectors, reusable insulin pen injectors, syringes with general-purpose needles, single-use syringes for prefilled drugs, and prefilled syringes with needles that obtained certification as medical devices. The investigation covers the period 2000–2024.

### 2.4 Classification and main certification criteria for Japanese medical device nomenclature

Using the PMDA's Medical Devices Certification Information list (13) (as of January 18, 2024), this study examined certification product classifications related to six Japanese medical device nomenclatures primarily associated with drug delivery devices. This investigation covered various related classification criteria such as drug type, administration method, single-dose administration/reusability, needle-attached/needle-free, manual/power-driven, and the main certification criteria.

• Reusable medication/vaccine injector
• Pen-type medication injector
• Reusable insulin pen injector
• Syringe with general-purpose needle
• Single-use syringe for prefilled drugs
• Prefilled syringe with needles

### 2.5 Japanese medical device nomenclature for the mechanical parts of drug delivery devices in suspected failures reports

Based on the Pharmaceutical and Medical Devices Act, the PMDA collects and publishes information on suspected failures related to the device components of combination drugs reported by manufacturing authorization holders. Using the “Case Reports of Suspected Failures, etc. related to the Device Component of Combination Drugs” (14) (case analysis from November 25, 2014, to January 31, 2024), autoinjectors were extracted by searching for products surveyed in Section 2.1 and the medical device nomenclatures of the device parts listed in “Names of Machinery and Equipment Parts” were researched. Products with no reported suspected failures were excluded, and if multiple “Names of Machinery and Equipment Parts” were reported for one product, each was counted.

## 3 Results

### 3.1 Number of drug delivery devices as combination drugs in the Japanese market

Based on the PMDA pharmaceutical information search database (10) (as of June 3, 2024), the number of marketed products was determined by examining the number of package inserts for each combination drug. The search was conducted by looking for package inserts that included device names, such as “syringe,” “pen,” and “autoinjector,” in the “packaging” section. Due to the lack of standardized naming conventions for device components, pen-type devices were classified as “pen injectors” for combination drugs using needle-free disposable devices for multiple administrations and as “autoinjectors” for combination drugs using single-use disposable devices with needles for single administration. Among the four drug delivery devices, prefilled syringes had the highest number of products, followed by autoinjectors, which had the second highest number of products. Currently, the number of autoinjectors is approximately three times that of the pen injectors (Table 1).

**Table 1:** Number of drug delivery devices as combination drugs in the Japanese market.

Drug delivery device	Number
Prefilled syringe	160
Pen injector	12
Autoinjector	34
On-body injector	3

### 3.2 Investigation of autoinjectors in the Japanese market

**Figure 1:**
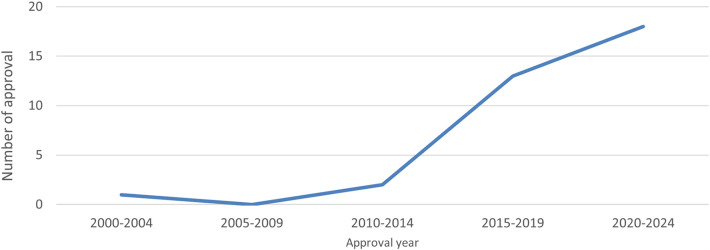
Annual number of approvals for autoinjectors.

This study investigated several drug delivery devices that use autoinjectors for combination drugs in Japan. The graph below shows the number of approvals of autoinjectors as combination drugs, including biosimilars, by approval year. It is evident that the number of approvals steadily increased from 2015 (Figure 1).

**Figure 2:**
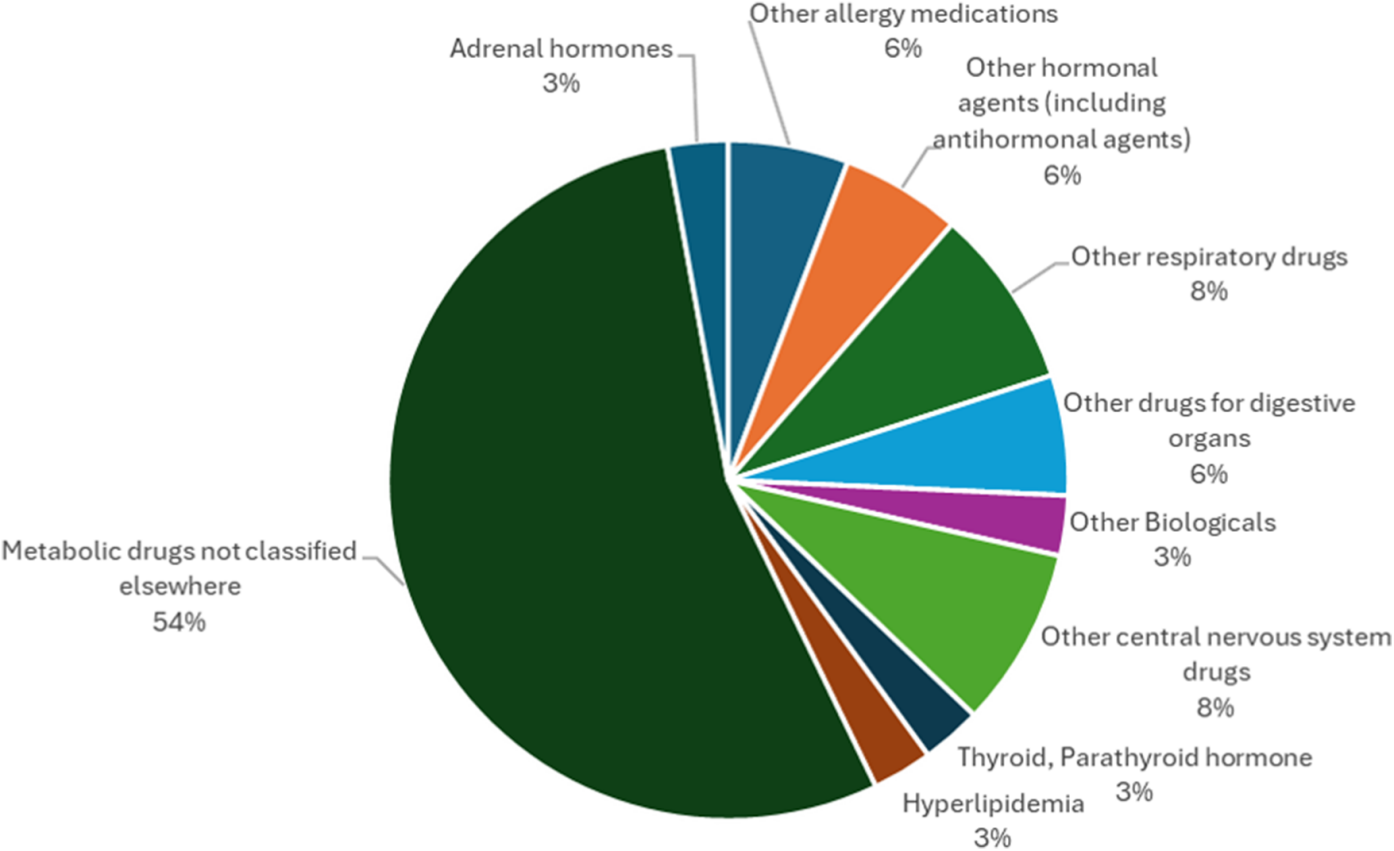
Efficacy classification of combination drugs using autoinjectors.

The results of this study regarding the efficacy classification of combination drugs using autoinjectors are presented below. The study on autoinjectors identified in Section 2.1 revealed that “Metabolic drugs not classified elsewhere” accounted for the largest number (54%), more than half of the total, followed by “Other respiratory drugs,” including those used for bronchial asthma, and “Other central nervous system drugs” used for suppressing the onset of migraine headache attacks, accounting for the same number. Although approximately half of the drugs in autoinjectors are “metabolic drugs not classified elsewhere”, including those for rheumatoid arthritis, there are a variety of other indications suggesting that they are used to treat a wide range of diseases (Figure 2).

**Figure 3:**
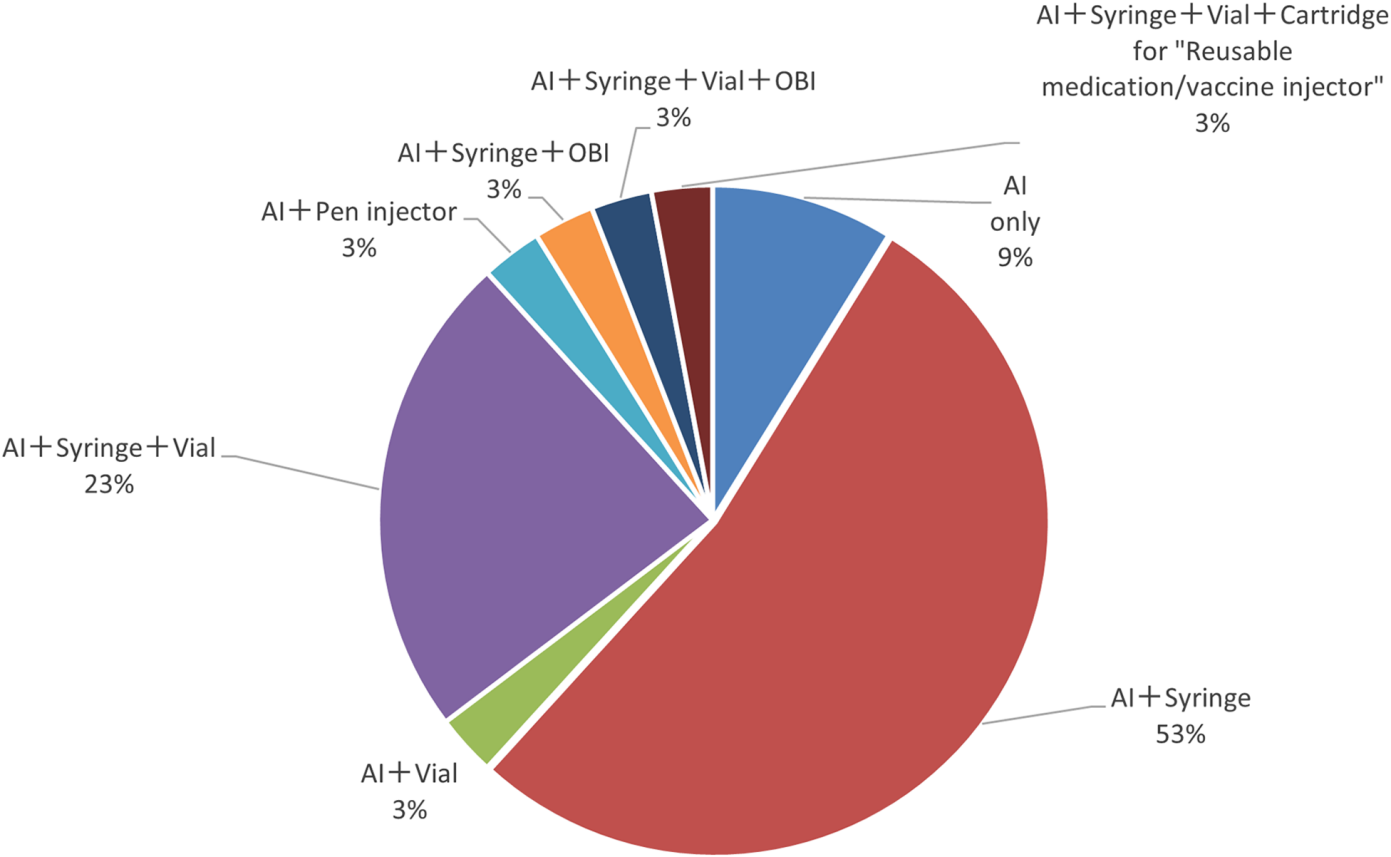
Approved formulations of combination drugs with autoinjector formulations.

Next, the study also found that a percentage of items among the autoinjectors surveyed had multiple formulation approvals besides the autoinjectors. The results showed that more than half (53%) of the items received approval as autoinjector and syringe, followed by a combination of the autoinjector, syringe, and vial (23%) (Figure 3).

**Figure 4:**
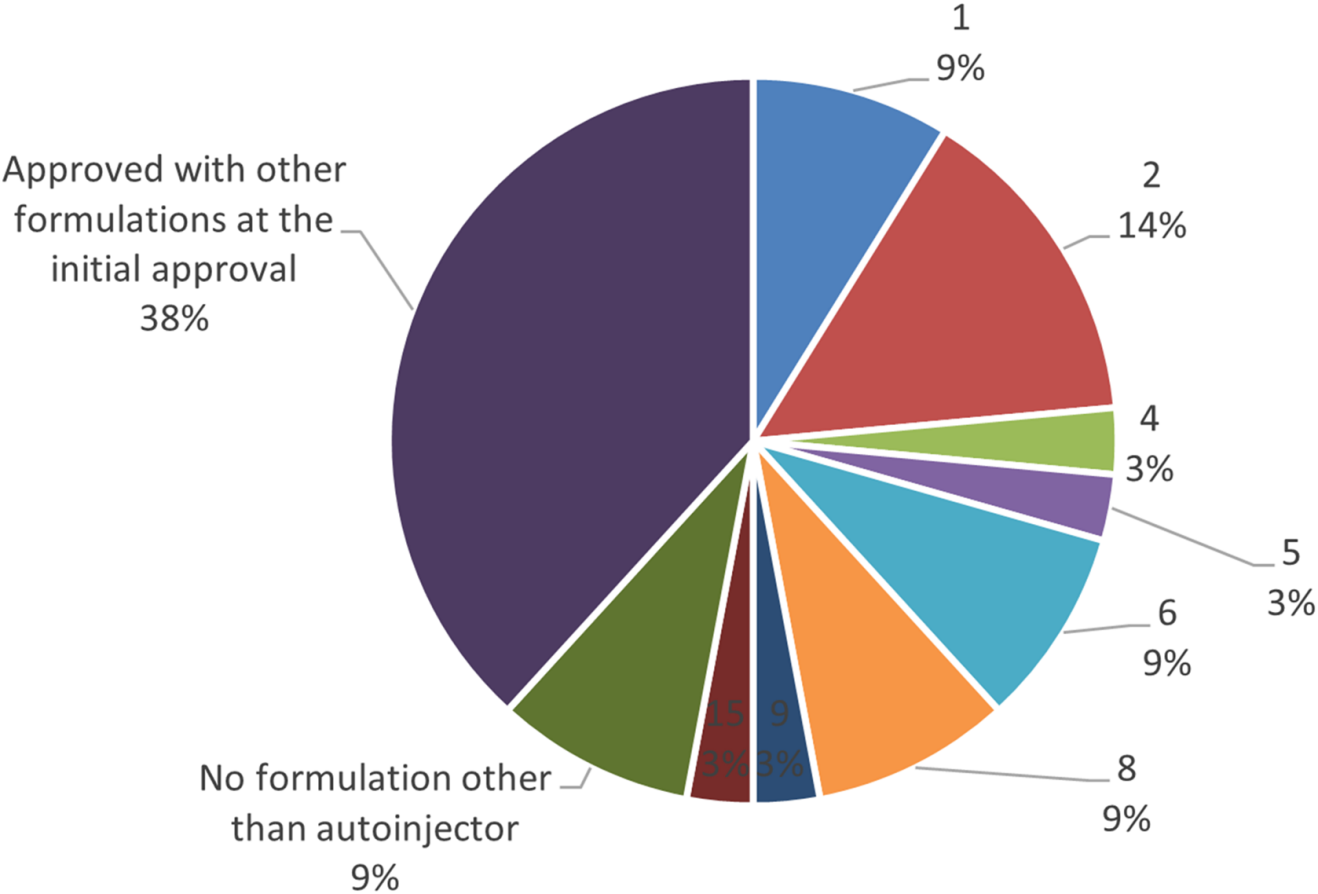
Number of years from the year of initial marketing approval to the year of autoinjector formulation approval (Pie chart).

**Figure 5:**
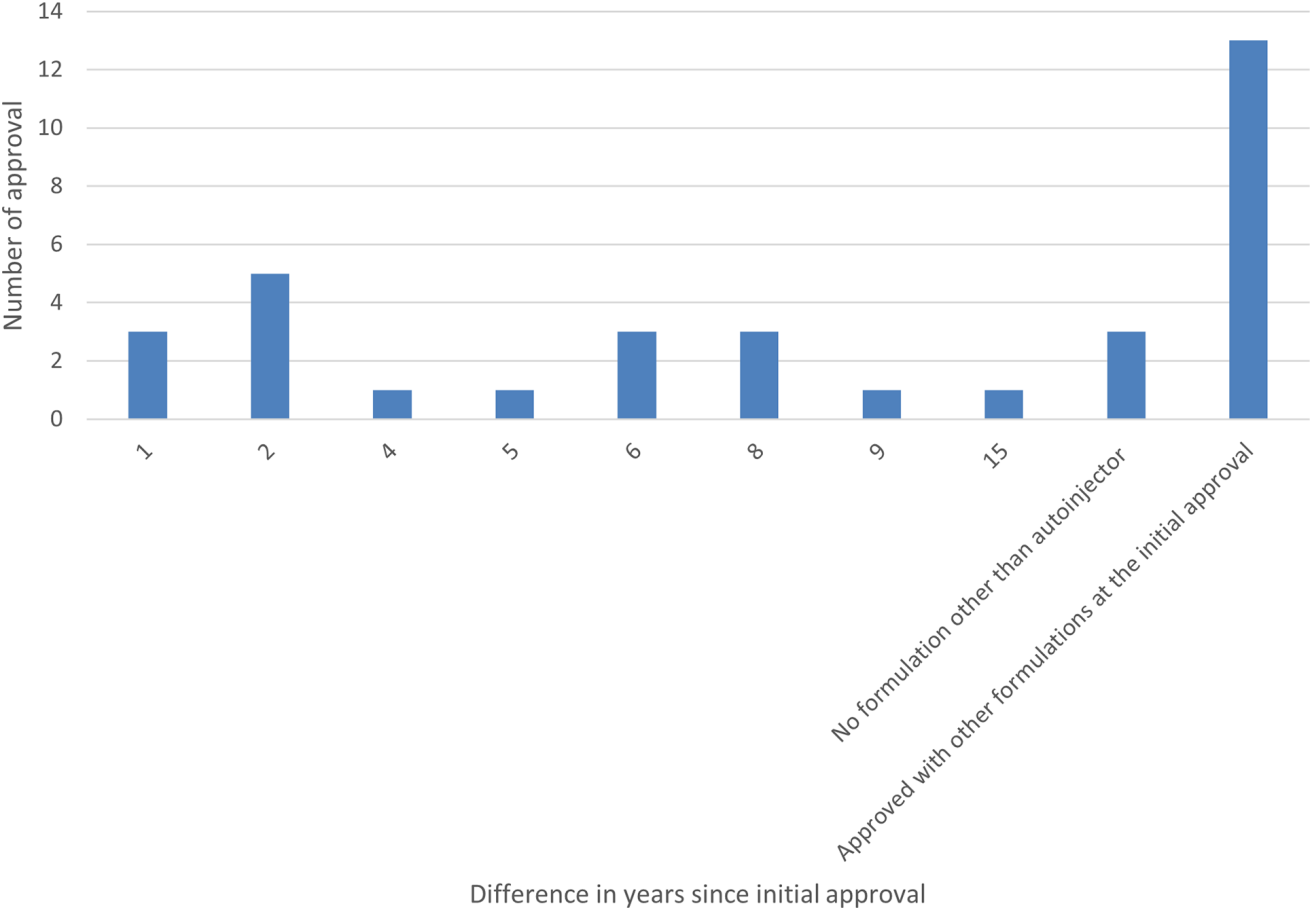
Number of years from the year of initial marketing approval to the year of autoinjector formulation approval (Bar graph).

Furthermore, the survey examined the percentage of autoinjector formulations approved since the initial marketing approval owing to additional formulations and the number of years from initial marketing approval to autoinjector approval. As a result, 38% of all items were approved as an autoinjector formulation in combination with other formulations at the time of initial marketing approval. Even when items with a single autoinjector formulation (9%) were combined with items that obtained an autoinjector formulation at the time of initial marketing approval (38%), less than half had an autoinjector formulation since the initial marketing approval. More than half (53%) of all items received approval for an autoinjector formulation since the initial marketing approval, and of these, approximately half or more received approval for an additional autoinjector formulation within five years of the initial marketing approval, with the largest number receiving approval for an additional autoinjector formulation within two years (14%). Even for combination drugs that obtained initial marketing approval after 2015, when the number of autoinjector launches began to increase, some drugs, such as DUPIXENT®, NUCALA®, Skyrizi®, Ozempic®, Metoject® took several years between initial approval and additional autoinjector formulation approval (Figures 4, 5).

### 3.3 Annual changes in the number of drug delivery devices approved and certified as medical devices and the number of canceled certifications

**Figure 6:**
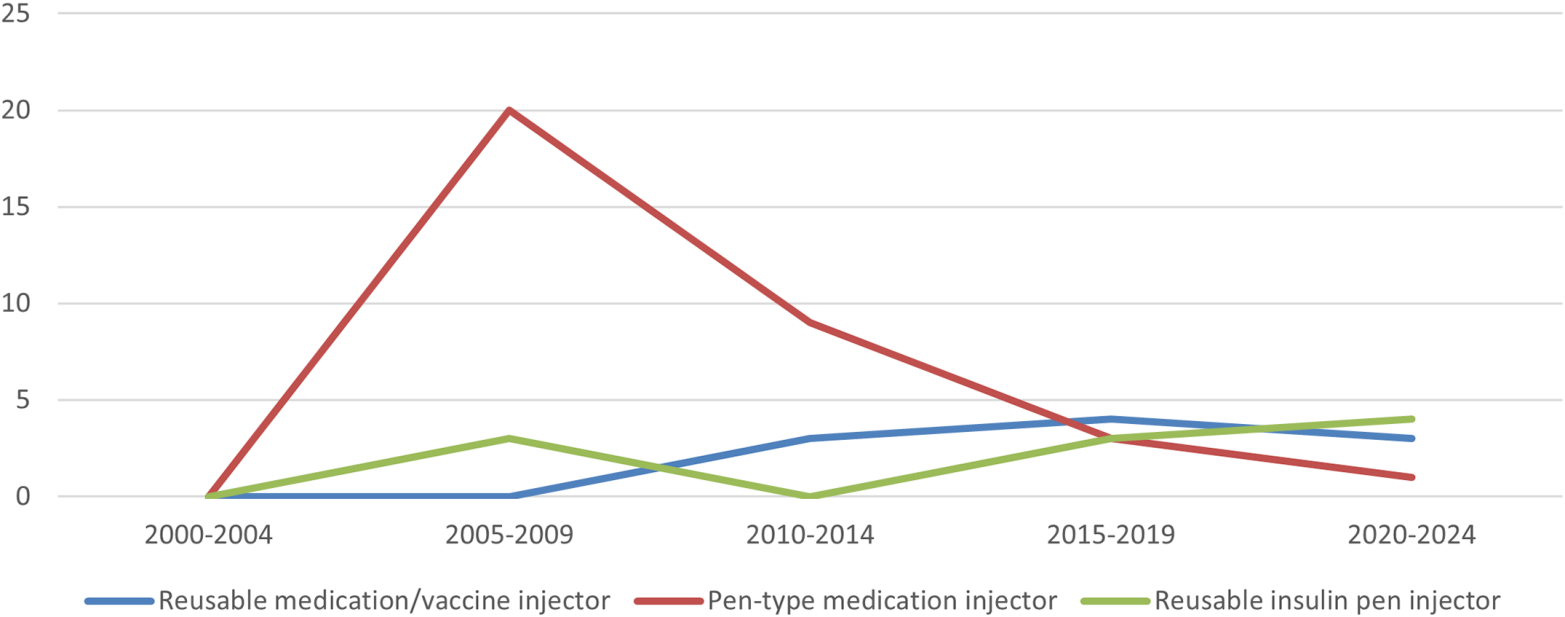
Number of approvals/certifications of drug delivery devices as medical device.

**Figure 7:**
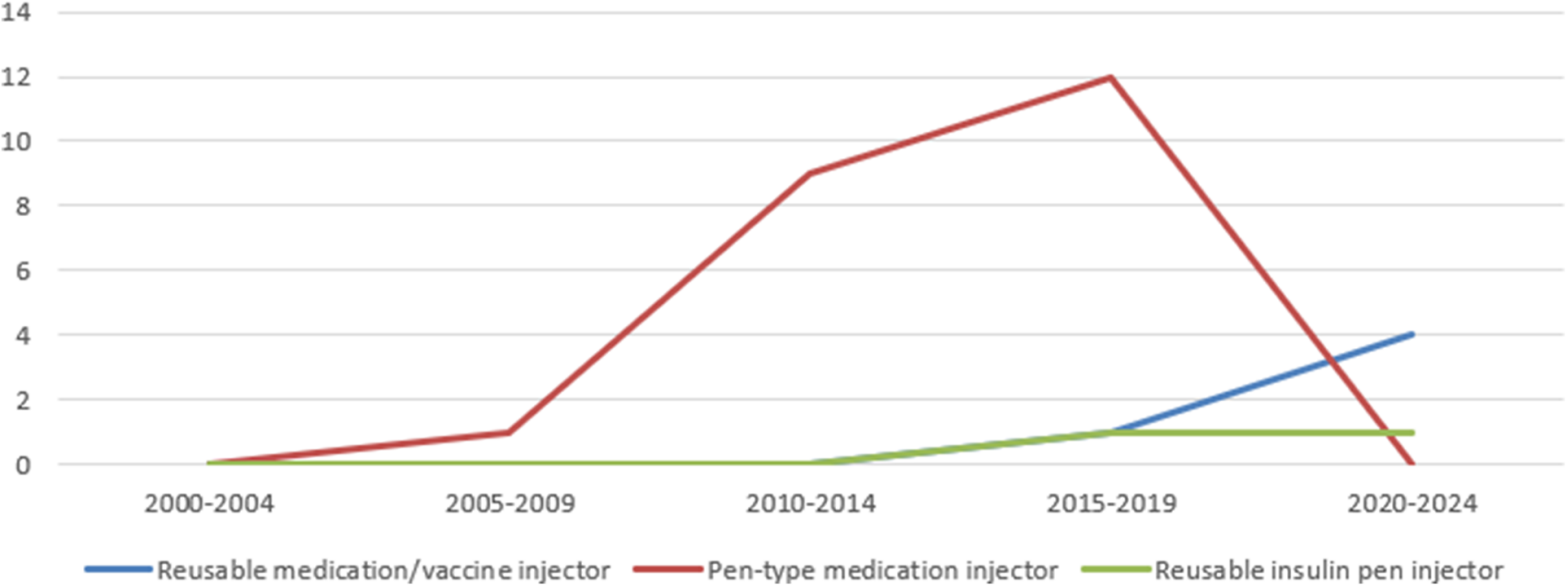
Number of canceled certifications of drug delivery devices as medical device.

Using the PMDA's Medical Device Information Search site (12) (as of June 9, 2024) and the certified product list (13) (up to the items certified in March 2024), the number of approved and certified reusable medication/vaccine injectors, pen-type medication injectors, and reusable insulin pen injectors that obtained approval/certification as medical devices from 2000 to 2024 was determined. The results showed that, of the three, pen-type medication injectors received the highest number of approvals/certifications to date. Pen-type medication injectors received the highest number of approvals/certifications between 2005 and 2009, and the number of approval certifications received has declined since 2010. Further investigation into the number of canceled certifications showed that the number of items canceled began to increase after 2010, with the number of cancellations peaking between 2015 and 2019. In the case of one reusable medication/vaccine injector for which the year of approval was unknown, the year of publication of the first package insert was counted (Figures 6, 7).

### 3.4 Classification of medical device certification by medical device nomenclature and main certification criteria

The following six certification items related to injectors are listed with their characteristics.

The device components of autoinjectors are essentially approved as combination drugs along with pharmaceuticals and are not additionally approved or certified as medical devices. Therefore, no established certification criteria or related quality standards for device components have been established (Table 2).

**Table 2:** Classification of medical device certification by medical device nomenclature and main certification criteria.

Japanese medical device nomenclature	Definition	Drug type	Method of administration	Single dose, reuse	With needle, without needle	Manual, powered	Main certification criteria
Reusable medication/vaccine injector	A device used for intramuscular (IM) or subcutaneous injection of drugs/vaccines into the human body. The device is usually reusable and comes in varying forms depending on the purpose. The device is either manually operated or powered (e.g., by spring, compressed gas, or electricity). The device differs from conventional syringes for subcutaneous injection. The device requires an injection needle for use. The devices used for injection of insulin are excluded.	drugs/vaccine (Excluding insulin)	intramuscular (IM) or subcutaneous	Reusable^a^	without needle	Manual or power type (e.g., by spring, compressed gas, or electricity)	JIS_T_0601–1
Pen-type medication injector	A manually operated pen-type device used for intramuscular or subcutaneous injection of drugs (excluding insulin) into the human body. The device is reusable (mostly pen-type), and each injection requires a new, replaceable needle tip to be attached. The structure varies depending on the purpose. The drug to be administered comes with the product (e.g., in a cartridge) and, depending on the purpose of the drug, is injected by medical stuff or the patient. The device is not a subcutaneous syringe.	Drugs (Excluding insulin)	Intramuscular (IM) or subcutaneous	Reusable	Without needle	Manual	JIS_T_3226-1
Reusable insulin pen injector	A manual device for subcutaneous insulin injection in humans. The devices are reusable (many are pen-type devices), and each injection requires a dedicated, replaceable needle tip to be attached. The device mechanism varies depending on the purpose. A cartridge, prefilled with insulin to be injected, is inserted in the pen, and the insulin is injected by a healthcare professional or the patient, depending on the circumstances. The device is not a subcutaneous syringe.	Insulin	Subcutaneous	Reusable	Without needle	Manual	JIS_T_3226-1
Syringe with general-purpose needle	A device used to inject or withdraw a fluid or gas. The device is normally made of glass or plastic and consists of a container with a scale and a plunger. The device is often used to administer drugs or collect blood.	Fluid or gas	N/A	N/A	With needle	N/A	JIS T 3209 JIS T 3210 ISO 80369-7 JIS T 3253
Single-use syringe for prefilled drugs	A glass or plastic syringe used to administer drug. Usually, the syringe is designed to contain single dose of drugs. This device is for single-use.	Drugs	N/A	Single dose	Without needle	N/A	N/A
Prefilled syringe with needles	A single-use glass or plastic syringe with a needle, used to administer drug. Usually, the syringe is designed to contain single dose of drugs,	Drugs	N/A	Single dose	With needle	N/A	JIS_T_0993-1

^a^ Not a requirement.

### 3.5 Japanese medical device nomenclature for the mechanical parts of drug delivery devices in suspected failures reports

When a combination drug containing a medical device that has not been individually approved and certified is approved, the general device-related information that is normally included in the application for approval and certification of the medical device is to be included in an attachment of the application for approval of the drug (15). Therefore, device-related information in public documents such as package inserts, interview forms, and review reports is limited, and it is difficult to obtain detailed device-related information.

**Figure 8:**
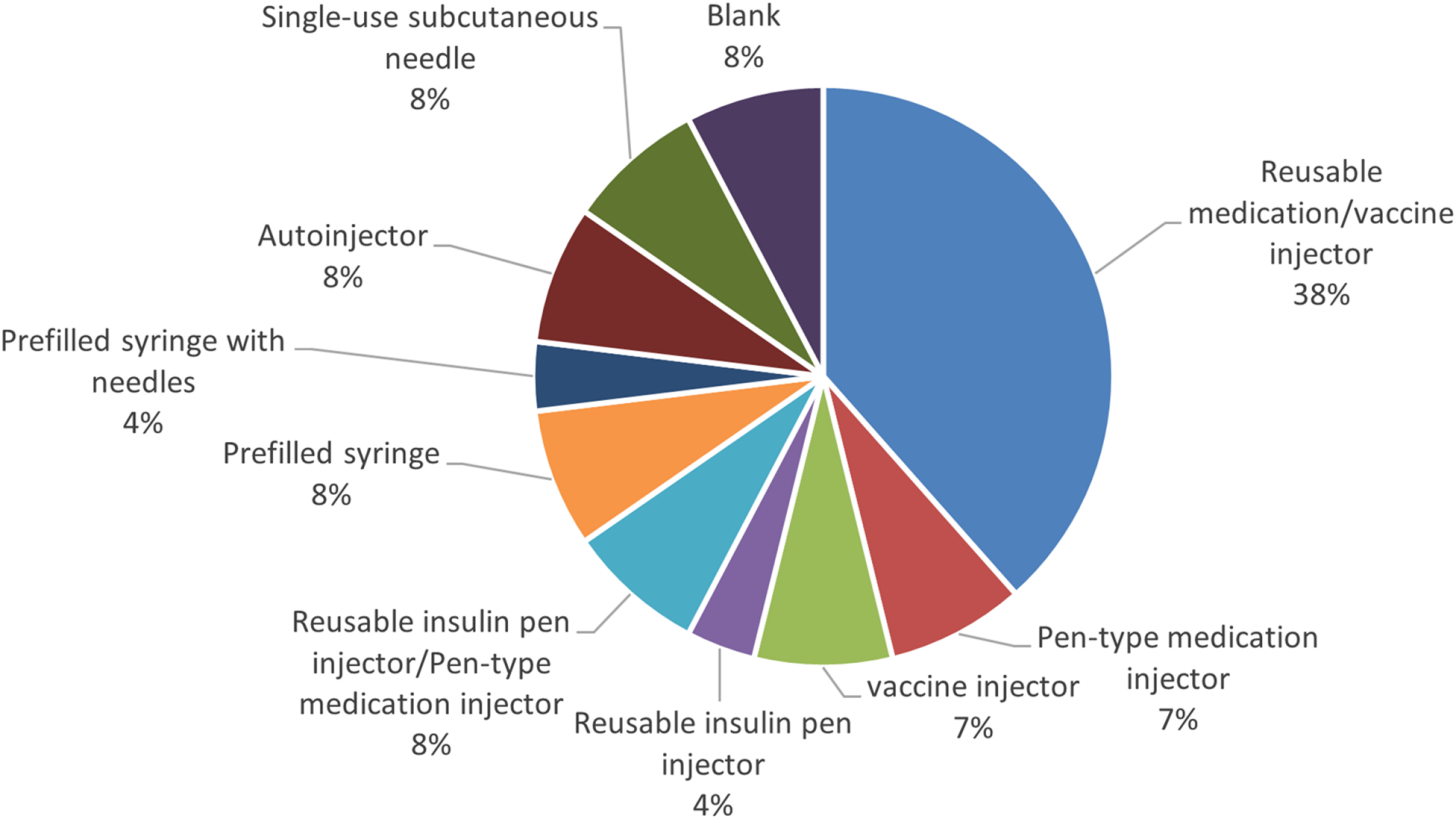
Reported name of device component of autoinjector in suspected failures reports.

In the past, the handling of adverse drug reactions and failure reporting for combination drugs were unclear, and failure reports for the prefilled syringe portion were not subject to reporting requirements. However, the Pharmaceuticals and Medical Devices Law, which came into effect on November 25, 2014, requires marketing authorization holders to report suspected failures in the device components of combination drugs. Currently, the PMDA collects and publishes information on cases of suspected device failure reported by marketing authorization holders for combination drugs. The names of the device components of autoinjectors were researched in the “Case Reports of Suspected Failures, etc. related to the Device Component of Combination Drugs” (14) (from November 25, 2014, to January 31, 2024) published by the PMDA. The results of the analysis after excluding items for which no cases of suspected failures were reported (17 out of 34 products) showed that the most common name of the device component of autoinjectors reported by marketing authorization holders was “Reusable medication/vaccine injector,” but a variety of names were used for each item, and there were some cases in which the name differed from the definition of a medical device nomenclature (Figure 8).

## 4 Discussion

### 4.1 Regulation of combination products in Japan

Combination products in Japan are defined as products that combine two or more types of drugs, medical devices, or cellular and tissue-based products that are expected to be considered drugs, medical devices, or cellular and tissue-based products when distributed separately. The current classification categorizes combination products into three types: (1) “Set Products” that can be distributed separately, (2) “Kit Products” that are integrated and cannot be distributed separately, and (3) “Products other than Kit Products that are integrated and cannot be distributed separately” (16).

### 4.2 Historical classification of combination products in notifications

In 1986, a Notification related to combination products was issued, but since the term “combination product” and the concept of cellular and tissue-based products did not exist, the treatment of products combining a drug and a medical device (including a container) or two or more drugs as a single administration system was indicated as “kit products.” At the time, pharmaceutical products and medical devices required separate applications for approval (17). In 2004, a pharmaceutical product was added to the kit product category as a container for inhalation (18). Subsequently, the Pharmaceutical and Medical Devices Act has been revised. In 2014, “cellular and tissue-based products” were defined, and the first notification of the current handling notice was issued, clarifying the term “combination product.” Depending on the main function and purpose, it was determined whether the main component of the product fell under the category of drug, medical device, or cellular and tissue-based products and whether the application was to be filed as a product that fell under that category. If the main component is a drug and the secondary component is a medical device, it is filed as a drug; filing an application as a medical device is not mandatory. If the secondary component is approved, information on the approval was to be stated as ingredients or components in the application for approval of the relevant combination product (8, 15). In 2016, it was suggested that combinations with what is defined as a container^1^ in the definition of a medical device are not covered by the combination product. However, “Prefill syringes,” which had previously been defined as containers, continued to be covered by the combination product since the definition of “container” was removed (19). The current notification on combination products clearly states that products combined with the above-mentioned “containers” are not covered by combination products and that kit products in which the drug product is filled inside a container for inhalation are not covered by combination products (15). (Inhalers with an inhalation volume adjustment function remain covered under combination products).

### 4.3 Trends in drug delivery devices approved and certified as combination drugs or medical devices

Among the various drug delivery devices used in combination drugs, such as prefilled syringes, pen injectors, autoinjectors, and on-body injectors, the number of autoinjectors is next to that of prefilled syringes, which has rapidly increased since 2015 (as of June 2024). However, the number of pen-type medication injectors approved or certified as medical devices has declined since 2005–2009. Several factors could explain this shift. The first is the regulatory background of the issuance of notification on combination products in 2014, which no longer requires a separate application for drug delivery devices as a medical device for combination drugs (8). Second, in terms of device function, disposable autoinjectors and disposable pen injectors offer the convenience of not requiring cartridge installation, and as the development of devices with improved usability progresses, autoinjectors are used for a wide range of therapeutic indications. Third, it is thought that drug makers are trying to differentiate their products from similar drugs of the same type, including both non-generic and generic drugs, by adding value not only in terms of drugs but also in terms of drug delivery devices. Development is underway to bring the same type of drug delivery device to the market so that they are not differentiated from each other. It can be inferred that the market needs are switching because of these factors.

### 4.4 Challenges in product development of autoinjectors

The problem with autoinjectors is the lack of uniformity in the naming conventions for their marketing names, as various formulations, such as “pen” and “autoinjector,” are mixed together, and some items do not have a name that identifies the formulation in the marketing name (9).

In addition, a study on combination drugs with autoinjector formulations revealed that only 9% of all cases had a single autoinjector formulation under a single brand name, and more than 90% had multiple formulations approved, including syringes and vials, in addition to the autoinjectors. However, less than half of all cases had an autoinjector formulation at the time of initial marketing authorization. Although it was originally considered beneficial to obtain approval for autoinjector formulations from the time of initial marketing approval because of their high usability and reduced patient burden, more than half of the cases were added after initial marketing approval as additional formulations, and the most common case was when the additional autoinjector formulation was approved two years after the initial marketing approval.

Because combination drugs with autoinjector formulations are filed as drugs, no criteria for certification as medical devices or any other quality standards are stipulated. In this investigation of suspected failure reports disclosed by the PMDA, using the name of the mechanical device component of the autoinjectors as an example, it was found that the recognition of medical device nomenclature differs among marketing application holders. This suggests there may be confusion among marketing application holders due to inconsistent recognition of the device components of combination drugs.

It is possible that pharmaceutical manufacturers intentionally delay the launch of combination drugs with autoinjector formulations owing to a lack of expertise and experience in devices or business judgment. However, the lack of clear guidelines from the regulatory authorities on strategies and quality standards for regulatory applications for combination drugs may have made it challenging to include autoinjector formulations in the initial marketing approval process because of concerns about the extended filing preparation and review period for approval. It may have delayed the launch of autoinjector formulations by several years compared to other formulations under the same brand name.

## 5 Recommendations

Several recommendations can be made to address these challenges and improve the regulation and development of combination drugs and drug delivery devices. Clear guidelines and requirements should be established for device components of combination drugs, including device-related regulatory filing processes, regulatory requirements, quality standards, and naming conventions. This would contribute to consistent product quality, reduce the burden on both regulatory health authorities and applicants, and enhance usability and convenience for users. Additionally, developing user-friendly product names and improving public documents, such as package inserts and interview forms, is essential.

Furthermore, in addition to drug manufacturers applying for combination drug approval for both drugs and devices, exploring the application of a Device Master File for the device components of combination drugs could facilitate the inclusion of device manufacturers' knowledge, streamline the application and review process, ensure uniformity of product quality, and allow for efficient post-market changes and improvements. This is because the device components of combination drugs have improved more quickly than those of drugs based on the latest findings, and the same type of device from the same device manufacturer is often used for multiple combination drugs.

## 6 Conclusions

The trend in the pharmaceutical and medical device industries is changing rapidly with the introduction of various drug delivery devices in recent years. In this study, an investigation was conducted on approved or certified combination drugs and medical device products, and it was found that the number of combination drugs with autoinjectors is increasing, replacing previously approved or certified pen-type medication injectors as medical devices. However, more than half (53%) of the products with autoinjector formulations obtained approval for the autoinjector formulation after the initial marketing approval, of which more than half obtained approval for an additional autoinjector formulation within five years of the initial marketing approval, with the largest number of cases obtaining approval for an autoinjector formulation within two years.

This indicates that the introduction of autoinjector formulations is often delayed compared with other formulations of the same brand, and more than half of the products did not obtain approval for the autoinjector formulation at the time of initial marketing approval. The lack of uniform recognition of device components among marketing authorization holders and the absence of clear regulatory requirements may contribute to confusion among applicants. The lack of clear regulatory requirements poses challenges to the regulatory process and hinders the market entry of combination drugs that utilize user-friendly devices. Access to more convenient and user-friendly devices is an important aspect of pharmaceutical treatment and significantly impacts patient adherence and treatment efficacy. Establishing clear regulatory processes, requirements, and quality standards for the device components of combination drugs in line with market trends is crucial for streamlining the review process, ensuring consistent product quality, and facilitating efficient post-market changes and improvements. This study is an attempt to identify market issues in regulatory filings for drug delivery devices in Japan. We believe the findings of this study are beneficial for the development strategies and innovation for drug delivery devices. We are also confident that they will facilitate improved patient access to more convenient devices at an early stage.

## 7 Limitations

In the case of combination drugs that use devices without approval or certification as medical devices, there is limited official information available in public documents, such as package inserts and review reports regarding device components. The lack of clear information on device components and the different naming conventions used by manufacturers may have resulted in an inability to accurately identify all combination drugs.

## Data Availability

The original contributions presented in the study are included in the article/Supplementary Material, further inquiries can be directed to the corresponding author.

## Footnotes

^1^The main component of a combination product is a drug/cellular and tissue-based product, and the secondary component is a general medical device, defined as a container in the Japanese medical device nomenclature.
